# A Technique of Deep Brain Stimulation of the Globus Pallidus Interna for Dystonia Under General Anesthesia With Sevoflurane

**DOI:** 10.7759/cureus.40819

**Published:** 2023-06-22

**Authors:** Mohammad AlMajali, Mayur S Patel, Niel K Patel, Justin K Zhang, Christopher Tapia, Richard D Bucholz, Pratap Chand

**Affiliations:** 1 Neurology, University of Iowa, Iowa City, USA; 2 Neurosurgery, The Ohio State University Wexner Medical Center, Columbus, USA; 3 Internal Medicine, University of California San Diego, San Diego, USA; 4 Internal Medicine, Saint Louis University School of Medicine, St. Louis, USA; 5 Neurosurgery, University of Utah, Salt Lake City, USA; 6 Neurology, Saint Louis University Hospital, Saint Louis, USA; 7 Neurological Surgery, Saint Louis University School of Medicine, Saint Louis, USA; 8 Neurology, Saint Louis University School of Medicine, Saint Louis, USA

**Keywords:** globus pallidus interna, general anesthesia, sevoflurane, dystonia, deep brain stimulation

## Abstract

Background

Globus pallidus interna (GPi) deep brain stimulation (DBS) is an established surgical procedure that confers a benefit in medication refractory dystonia. Patients with generalized dystonia require general anesthesia (GA) for the surgery as their movements may hinder the surgical procedure. General anesthetics tend to dampen the microelectrode recordings (MERs) from the GPi.

Methods

We describe our experience with a series of consecutive patients with dystonia who underwent bilateral GPi DBS using standard DBS and MER under GA using sevoflurane as the maintenance general anesthetic drug. All patients had Medtronic 3,387 leads implanted and connected to an RC battery. Patients underwent sequential programming of the DBS after the surgery.

Results

The mean age of the 13 patients who underwent DBS of the GPi for dystonia was 46.5 years with a range from 29 to 71 years. Every patient in our case series received various doses of (1.37% to 2.11%) inhaled sevoflurane for anesthesia maintenance. Sevoflurane provided adequate anesthesia and allowed accurate MERs from the GPi. No adverse effects were encountered. On follow-up and sequential DBS programming, patients had significant improvements in dystonia attesting to the accuracy of the electrode placements.

Conclusions

We report our experience using sevoflurane for maintenance of GA for bilateral GPi DBS for dystonia. The main benefits identified have been adequate anesthesia and reduction of dystonia-related movements to allow the performance of the DBS surgery. The MER signals from the GPi were not suppressed by sevoflurane. This allowed accurate mapping and placement of the DBS implants in the GPi.

## Introduction

Deep brain stimulation (DBS) is an established neurosurgical procedure consisting of microelectrode recording (MER)-based targeting and placement of permanent electrodes followed by stimulation and neuromodulation of specific deep nuclei in the brain [1]. Dystonia is a broad spectrum of neurological movement disorders defined by involuntary muscle contractions resulting in repetitive sustained movements and modified posturing [2]. Advancements in the precision of DBS have introduced globus pallidus interna (GPi) stimulation as a viable first-line treatment option for medication-refractory dystonia [2,3].

Traditionally, DBS is performed under local anesthesia (LA) and conscious sedation to ensure the high quality of MERs obtained during the procedure and to ensure precision in electrode placement [4]. However, given the presence of frequent, spontaneous movements in patients with severe dystonia, surgeons may be required to utilize general anesthesia (GA) to be able to perform the surgical operation. Previous studies suggested that DBS under GA for patients with severe movement symptoms secondary to dystonia has resulted in similar levels of clinical improvement when compared to DBS performed under LA [5,6]. In addition to clinical improvement, further studies have demonstrated that the quality of MER and the accuracy of mapping the GPi (the target for dystonia) may be preserved under GA [7,8].

Despite these findings, evidence supporting specific GA medications in the context of DBS for dystonia remains limited. One study performed by Malekmohammadi et al. supported the use of propofol as part of the GA regimen, as it was found to maintain the precision of intraoperative recordings and result in equal symptomatic improvement in patients [9]. The following case series examines data from 13 consecutive patients over a period of 13 years (2009 to 2021) to demonstrate the use of a sevoflurane anesthesia protocol as a safe and effective option that preserves surgical accuracy of GPi targeting and mapping for patients undergoing DBS for dystonia.

## Materials and methods

Patient selection

We conducted a single-center retrospective case series of patients with dystonia who underwent bilateral DBS of the GPi under GA. Data collection was approved by the Institutional Review Board of our institution with a waiver of patient consent. All patient information was de-identified. Thirteen consecutive patients were selected from 2009 to 2021 who underwent this procedure with sevoflurane for maintenance GA. The cases presented were performed by a single functional neurosurgeon (RB) and the intraoperative MER and later programming of the DBS were performed by a single movement disorders neurologist (PC) at Saint Louis University Hospital. In all of the patients, informed consent was obtained, and the DBS was performed under an HDE IRB protocol. All patients had Medtronic 3,387 leads implanted and connected to a rechargeable (RC) battery.  Every patient in our case series received sevoflurane for anesthesia maintenance.

**Table 1:** Demographics and clinical features of dystonia

Characteristics		
No. of patients	13	
Gender	Percentages (n)	
Male	54 (7)	
Female	46 (6)	
	Mean	Range
Age (years)	46.5	29-71
Symptom length (years)	16.5	3-49
Dystonia	Percentages (n)	
Generalized	23 (3)	
Focal	15 (2)	
Multifocal	38 (5)	
Segmental	23 (3)	

Surgical procedure and MER

The surgical technique for DBS was standardized for all patients in this study. The targets were bilateral GPi. The DBS electrode trajectory was planned with standard stereotactic coordinates using a frameless system (Nexframe Stereotactic system using Stealthstation S7, Medtronic, Minneapolis, USA) and intraoperative O arm CT and confirmed with adjunct verification by MER. The electrodes were moved in intervals of 0.5 to 1 mm along the trajectory based on the MER to reach the appropriate targets. Overall, we followed the typical DBS of the GPi protocol for dystonia. Test stimulation for efficacy and adverse effects were not performed as the patients were under GA.

Single channel electrode MER of the GPi externa and interna were performed using a Lead point machine from 2009 to 2017 and from 2018 with the NeuroNav machine (Alpha Omega, Israel). Recordings were performed with appropriate microelectrodes and the GPis were mapped. All of the operations were performed under the supervision of RB and PC. All patients had bilateral GPi DBS. The surgery was performed on one side first and then the second side and later the leads were connected to the Medtronic RC battery in one session. Intraoperatively, the microelectrodes were advanced using the microdrive and the trajectory of the electrodes was observed by intraoperative CT scans. MER was obtained at appropriate distances from the GP pallidus externa, GP interna externa (GPie) and the GPi interna interna (the target) following previously described electrophysiologic morphology and frequency criteria [10]. The microelectrode was further advanced into the optic tract and photic stimulation was done through closed eyelids with a flashlight with recording of the response from the optic tract. All these recordings were possible despite the GA. Following this the microelectrode was brought up using the microdrive and located at the depth corresponding to the mapped GPi interna. The microelectrode was then removed, and the Medtronic 3,387 lead was implanted into the GPi interna with the first contact in the ventral GPi and secured. Intraoperative and later postoperative CT scans confirmed accurate placement. Test stimulation for efficacy and adverse effects could not be done because the patients were under GA. The MERs were all evaluated by RB and PC.

Anesthesia

All patients were induced with propofol, intubated and ventilated, and received sevoflurane during the procedure. Every patient in our case series received sevoflurane for anesthesia maintenance. We further reviewed anesthesia data including medications used for induction and maintenance, mean doses and expired concentration of sevoflurane, duration of induction, and total doses. The total lengths of the surgeries were also obtained.

Data and statistical analysis

A descriptive analysis was chosen for reporting data to account for differences between individual cases. We calculated the mean and ranges of the MER of the right and left GPI at appropriate distances from the target site as well as the signals at the target. The mean and range of dosages and percentages for the induction and maintenance anesthesia medication were also calculated. For inhaled medications (sevoflurane), the percentage of expired medication was also evaluated.

## Results

Patient characteristics  

We evaluated the medical records of 13 consecutive patients who underwent DBS for medication refractory dystonia. The mean age of the patients who underwent DBS of the GPi for dystonia was 46.5 years with a range from 29 to 71 years. Out of our sample, seven patients (54%) were male and six were female. On average, the patients were symptomatic for 16.5 years, ranging from three to forty-nine years. Apart from two, all patients presented with dystonia affecting multiple areas of the body. In our case, patients most commonly presented with multifocal dystonia (n=5). Our sample also included three patients with generalized and three patients with segmental dystonia. Only two patients had a focal dystonia presentation (Table 1).  Demographic information including age, gender, onset and length of symptoms, location of dystonia, and follow-up results for the 13 patients are outlined in Table 1.

Operative characteristics  

The mean length of operation for these bilateral surgeries was 598 minutes (range 500-704 minutes). There were no intraoperative or postoperative complications and all patients reported partial symptomatic improvement after their first programming visit usually four weeks after the surgery. The DBS leads were programmed four weeks after the surgery by PC. Six patients reported a slight improvement, two reported moderate improvement, and seven reported marked improvement. Their symptoms of dystonia continued to improve with monthly repeat programming sessions reaching a peak benefit at six months. Most followed up at six months thereafter and over the years small adjustments in current strength were required to control the symptoms. Improvements were maintained over the years of follow up.

Anesthesia regimens

The dosages of sevoflurane ranged from an average of 1.37% to 2.11% inhaled. Additionally, the percent of sevoflurane expired ranged from an average of 1.21% to 2.27%. The mean duration of sevoflurane administered was 420 minutes.

Patients at our institution also received various induction and maintenance medications in addition to sevoflurane. The mean dosage of propofol administered was 249mg (n=9; range: 100-520mg), remifentanyl was 2.5mg (n=7; range: 1.01-4mg), rocuronium was 58mg (n=6; range: 30-100mg), dexmedetomidine was 218mcg (n=5; range: 14.88-453.19mcg), fentanyl was 185mcg (n=5; range: 50-300mcg), and succinylcholine was 97mg (n=6; range: 80-100mg). The most common induction protocol used was propofol, remifentanyl, rocuronium, dexmedetomidine and maintenance was with sevoflurane.

## Discussion

GPi stimulation via DBS is an FDA-approved treatment option for individuals with medication-refractory generalized dystonia. GA is an emerging option for patients undergoing DBS with movement symptoms too severe to be adequately controlled intraoperatively via LA and conscious sedation. Despite advancements with GA, DBS-specific regimens with sevoflurane alone have not been clearly reported in the literature. We report a large case series of patients undergoing GPi DBS for dystonia using sevoflurane as the main maintenance general anesthetic agent.   

All patients in our case series received maintenance sevoflurane during the recording of MER with mean inhalation concentrations adjusted for patient weight. Remifentanyl and Propofol were the most common additional agents used for induction of anesthesia and remifentanyl was the commonest agent used along with Sevoflurane for maintenance during the procedure. The use of the MER has been found to be very useful for accurate targeting and electrode implantation for DBS [10-12]. In our institution, we have used it consistently for accurate electrode placement in the GPii. We also ensured that the path of the electrodes matched the planned trajectory using intraoperative O arm CT imaging.  

The visual and audio characteristics of the MER signals recorded show that sevoflurane provided adequate anesthesia while maintaining the quality of the MER obtained as compared to MER data obtained in our institution utilizing LA and conscious sedation and also from other studies [12,13]. The MER data obtained from patients who underwent our GA regimen is similar to previously reported data from patients under various GA regimens where the medications may vary by patients, institutions, and anesthesiologists' preferences [8,14]. The goal of our study is to describe our maintenance regimen with sevoflurane for GA. We propose that after induction of GA with propofol, dexmedetomidine, remifentanyl, the maintenance regimen should be with sevoflurane alone till the end of the procedure.  

In addition to the intraoperative feasibility of using sevoflurane for GA, our patients reported improvement in their dystonia symptoms following GPi DBS and seven out of 13 patients reported a greater than 50% reduction in dystonia with sequential DBS programming. These rates of clinical improvement are similar to previous studies found in the literature [15-18]. This demonstrates that our technique of MER mapping of the GPi was not adversely affected by the GA regimen and ensured good outcomes.  

Prior literature on the use of GA for DBS for dystonia has shown similar post-operative outcomes and accurate implant placements compared to LA [14,19]. Information on the exact medications and dosages administered is sparse in the literature with some studies reporting the use of GA without clearly describing the regimens [17,20].  Maintenance medications most commonly mentioned in previous studies include propofol, remifentanyl, desflurane, and sevoflurane [8,14,18]. Our current case series provides more comprehensive and specific data on sevoflurane and outcomes for 13 patients.  

The benefits of GA have been briefly outlined in the literature previously. Wang et al. reported a systematic review of 341 patients who underwent DBS under GA. Their results also indicate that this can be a safe and efficacious alternative to performing DBS with LA [6]. Another study by Li et al. reported clinical outcomes and Burke-Fahn-Marsden Dystonia Rating Scale (BFMDRS) scores in patients undergoing DBS of the GPi and subthalamic nucleus under GA. Their results show that GA can be an effective and safe method for anesthesia for DBS in patients with isolated dystonia [18]. The use of sevoflurane and desflurane has been reported in the literature for DBS for the subthalamic nucleus in Parkinson’s disease [19]. A previous study reported that a combination of anesthetics including low-dose propofol infusion, remifentanil, and a low concentration of either sevoflurane or desflurane provided better recordings of pallidal MER signals [8].

Limitations

Although our results demonstrate that sevoflurane does not hinder MER from GPi, our results are limited. We have a low sample size of 13 patients at a single center. We also do have a control group as the design of this study does not allow for comparing our cohort with a group with other anesthesia regimen. Although our results are promising, a larger and controlled investigation needs to a conducted for definitive conclusions.

## Conclusions

We report our experience using sevoflurane for maintenance of GA for bilateral GPi DBS for dystonia. The main benefits identified have been adequate anesthesia and reduction of dystonia-related movements to allow the performance of the DBS surgery. The MER signals from the GPi were not suppressed by sevoflurane. This allowed accurate mapping and placement of the DBS implants in the GPi. Furthermore, we report our observation that sevoflurane is safe and effective as a GA agent for bilateral GPi DBS for dystonia.
